# A Case of Retention of Urine Caused by a Stone in the Bladder

**Published:** 1885-09-20

**Authors:** G. E. Flowers

**Affiliations:** Georgia


### A CASE OF RETENTION OF URINE CAUSED BY A STONE IN THE BLADDER.

---

BY G. E. FLOWERS, M. D., OF GEORGIA.

---

I was called, May 10, 1880, to see a man sixty-four years old, a farmer, who had passed but little urine during upwards of four days. He told me he wanted something to operate on the bowels, as he had failed with oil, salts, pills, etc.; that he had passed a little urine every day; that his bowels were swollen and painful. On examination, I found his trouble to be an immensely distended bladder. He was quite despondent of any help, as injections were discharged as they were made. After great hesitation on his part (as he did not think the bladder was full), and persuasion from me, he allowed me to introduce a soft rubber catheter. I drew, before removing the catheter, nine pints of water. His bowels at once evacuated, his pains subsided, and he went to sleep.

I report this case simply because the young physician very seldom meets a case in which there is such a great amount of urine causing this dangerous distention of the bladder. The patient is still using the catheter, operating for himself every twenty-four hours.

